# The physical and cellular mechanism of structural color change in zebrafish

**DOI:** 10.1073/pnas.2308531121

**Published:** 2024-05-28

**Authors:** Dvir Gur, Andrew S. Moore, Rachael Deis, Pang Song, Xufeng Wu, Iddo Pinkas, Claire Deo, Nirmala Iyer, Harald F. Hess, John A. Hammer, Jennifer Lippincott-Schwartz

**Affiliations:** ^a^Weizmann Institute of Science, Department of Molecular Genetics, Rehovot 7610001, Israel; ^b^HHMI, Janelia Research Campus, Ashburn, VA 20147; ^c^Cell and Developmental Biology Center, National Heart, Lung, and Blood Institute, NIH, Bethesda, MD 20892; ^d^Weizmann Institute of Science, Department of Chemical Research Support, Rehovot 7610001, Israel

**Keywords:** structural colors, guanine crystals, zebrafish, color change, motor proteins

## Abstract

Many animals exhibit remarkable colors that are produced by the constructive interference of light reflected from arrays of intracellular guanine crystals. These animals can fine-tune their crystal-based structural colors to communicate with each other, regulate body temperature, and create camouflage. While it is known that these changes in color are caused by changes in the angle of the crystal arrays relative to incident light, the cellular machinery that drives color change is not understood. Here, using a combination of 3D focused ion beam scanning electron microscopy (FIB-SEM), micro-focused X-ray diffraction, superresolution fluorescence light microscopy, and pharmacological perturbations, we characterized the dynamics and 3D cellular reorganization of crystal arrays within zebrafish iridophores during norepinephrine (NE)-induced color change. We found that color change results from a coordinated 20° tilting of the intracellular crystals, which alters both crystal packing and the angle at which impinging light hits the crystals. Importantly, addition of the dynein inhibitor dynapyrazole-a completely blocked this NE-induced red shift by hindering crystal dynamics upon NE addition. FIB-SEM and microtubule organizing center (MTOC) mapping showed that microtubules arise from two MTOCs located near the poles of the iridophore and run parallel to, and in between, individual crystals. This suggests that dynein drives crystal angle change in response to NE by binding to the limiting membrane surrounding individual crystals and walking toward microtubule minus ends. Finally, we found that intracellular cAMP regulates the color change process. Together, our results provide mechanistic insight into the cellular machinery that drives structural color change.

## Significance

Our study reveals the intricate cellular machinery underlying structural color change in animals, as demonstrated through our investigation of the zebrafish iridophore. By employing advanced imaging techniques and pharmacological perturbations, we have elucidated the role of dynein, microtubules, and intracellular signaling in the dynamic reorganization of crystal arrays and subsequent color modulation. These findings shed light on the mechanisms by which zebrafish achieve their remarkable color adaptations. Future investigations are essential to ascertain whether similar mechanisms are employed by other organisms with crystal-forming capabilities, particularly in relation to thermoregulation, camouflage, and social interactions. This work expands our knowledge of the fundamental principles governing biological coloration and has implications for the design of bio-inspired materials and devices with tunable optical properties.

The ability of animals to change their color has captivated scientists and naturalists for centuries (1–3). Animals change their color for numerous reasons, including thermoregulation, communication, camouflage, and protection from UV radiation (4–7). For animals whose color is pigment based, these color changes are classified as either morphological or physiological, depending on their mechanism and speed (4). Morphological color changes rely on the relatively slow production, degradation, or chemical modification of pigments, and are generally completed within hours to days (e.g., tanning). Physiological color changes, on the other hand, occur on the order of seconds to minutes (6, 8). Importantly, the mechanism responsible for such abrupt changes in pigment-based coloration has been studied extensively using fish melanophores, frog xanthophores, and frog erythrophores (9, 10), all of which exhibit rapid, hormone-controlled changes in animal color. This is accomplished via the rapid, microtubule-dependent repositioning of preexisting pigment granules (8, 11–15), with kinesin driving pigment dispersion to create a dark animal and dynein driving pigment aggregation to create a light animal. Manipulating the spatial distribution of light-absorbing pigments in this way controls the reflectance or scattering of ambient light, resulting in rapid and dramatic changes in animal color.

A second major form of animal coloration known as structural coloration involves the reflection or scattering of light by transparent nanostructures, which creates coloration by light interference (16). In guanine crystal-forming iridophores, which are abundant in both vertebrates and invertebrates (17–23), light reflectivity is the result of multilayer, thin film interference. This interference is created by alternating nanometric layers of high and low refractive index arising from membrane-enclosed guanine crystals and the cytoplasm, respectively (24). Changes in reflected color are achieved primarily by changes in the distances between the guanine crystals (25). The architectural changes that result in changes in crystal distance are known for certain species of fish (26–29) and have recently been described in copepods (30, 31) (planktonic crustaceans) and chameleons (32). That said, the cellular machinery and the molecular mechanisms responsible for color change in structure-based systems remain largely unknown. Here we used zebrafish scale iridophores as a model system to address the mechanism of structural color change in response to NE. Using a combination of 3D focused ion beam scanning electron microscopy (FIB-SEM) and micro-focused X-ray diffraction, we show that NE-induced color change results from a coordinated 20° tilting of the intracellular crystals, which alters both crystal packing and the angle at which impinging light hits the crystals. Imaging of crystal dynamics using a dye, mapping the distribution and orientation of microtubules with respect to the crystals, and application of pharmacological perturbations together argue that the microtubule minus end-directed motor dynein drives the NE-induced change in crystal angle by binding to the crystal’s limiting membrane and walking toward microtubule minus ends. Finally, we show that intracellular cAMP regulates the color change process. Together, our results provide mechanistic insights into the cellular machinery underlying structural color change.

## Results

### Iridophores within Isolated Scales Change Color in Response to Norepinephrine.

**Fig. 1.**
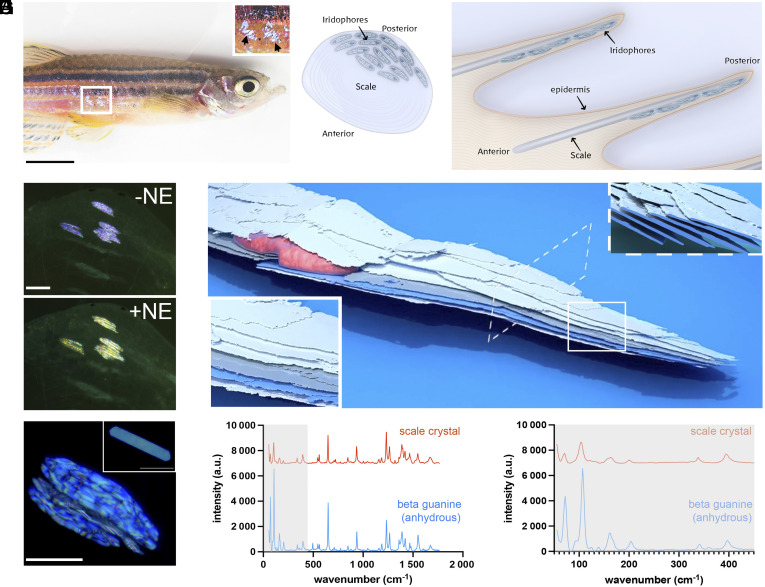
Anatomy, crystal properties, and color change capabilities of the zebrafish scale iridophores. (*A*) The flank of an adult zebrafish. The *Top Inset* shows a closeup of the reflectance of individual scale iridophores (marked with black arrows). (*B* and *C*) Schematic illustration of a zebrafish scale and tissue showing a *Top* view of an isolated scale in which iridophores are located adjacent to the scale on its posterior side (*B*), and a side view (cross-section) showing the scales within the fish dermis in which iridophores are located adjacent to the scales (*C*). (*D*) Scale iridophores before (*Top*) and after (*Bottom*) NE-treatment display violet-blue and yellow reflectance, respectively. (*E*) 3D rendering of FIB-SEM serial-sectioning of a scale iridophore showing an array of thin intracellular crystals arranged in parallel (highlighted by pseudocoloring in blue and gray; the cell’s nucleus is pseudocolored red). The *Bottom Left Inset* shows a higher magnification view of the parallel crystal arrays, while the *Top Right Inset* shows a perpendicular view of the crystal arrays. (*F*) Detected reflectance (blue) under the light microscope from scale iridophores. The *Top Inset* shows a single crystal isolated from a scale iridophore (*G*) In situ micro-Raman spectra obtained from scale iridophores (red spectrum), together with a reference spectrum for anhydrous β guanine (blue spectrum). (*H*) The low-wavenumber region of the samples examined in (*G*). (Scale bars, (*A*) 3 mm, (*D*) 80 µm, (*F*) 40 µm.)

Zebrafish skin stripes change color from blue to yellow in response to the stress hormone NE (33). Zebrafish skin pattern contains two anatomically separated populations of iridophores, one in the skin and one within scales that reside just beneath a transparent mineralized collagen matrix (Fig. 1 *A–C*). To investigate the mechanism of color change we used scale iridophores, which are much more optically accessible than skin iridophores. First, we asked whether iridophores within isolated scales, which are separated from the fish’s nervous system, change color in response to NE. To test this, we bathed isolated fish scales in 10 μM NE solution and recorded their color over time. Upon NE treatment, scale iridophores did indeed change their color from indigo blue to yellow over a period of several minutes (Fig. 1*D*), indicating that scale iridophores themselves are capable of sensing and responding to NE.

To better visualize the intracellular organization of iridophores from the micron to nanometer scale, we used FIB-SEM. In this technique, a focused beam of ions is used to mill away the surface of the sample while a scanning electron microscope simultaneously captures images of each exposed surface. The processed image stacks yield an isotropic, 8 nm-resolution 3D reconstruction that reveals the iridophore’s ultrastructure and the arrangement of crystals within it (Fig. 1*E* and Movies S1 and S2). Scale iridophores were found to be densely populated with stacks of inclined, evenly spaced, ~20 to 30 nm thick crystals running roughly in parallel and aligned with the cell’s long axis (Fig. 1*E*). To further evaluate the morphology and molecular identity of these crystals we used incident light microscopy and micro-Raman spectroscopy of individual cells. We found that the crystals present within the scale iridophores are much larger than the crystals present within skin iridophores (33) (Fig. 1*F*) and showed a typical Raman spectrum for β-anhydrous guanine crystals (Fig. 1 *G* and *H*).

### Significant Changes in Crystal Tilt and Spacing Occur upon NE-Induced Color Change.

**Fig. 2.**
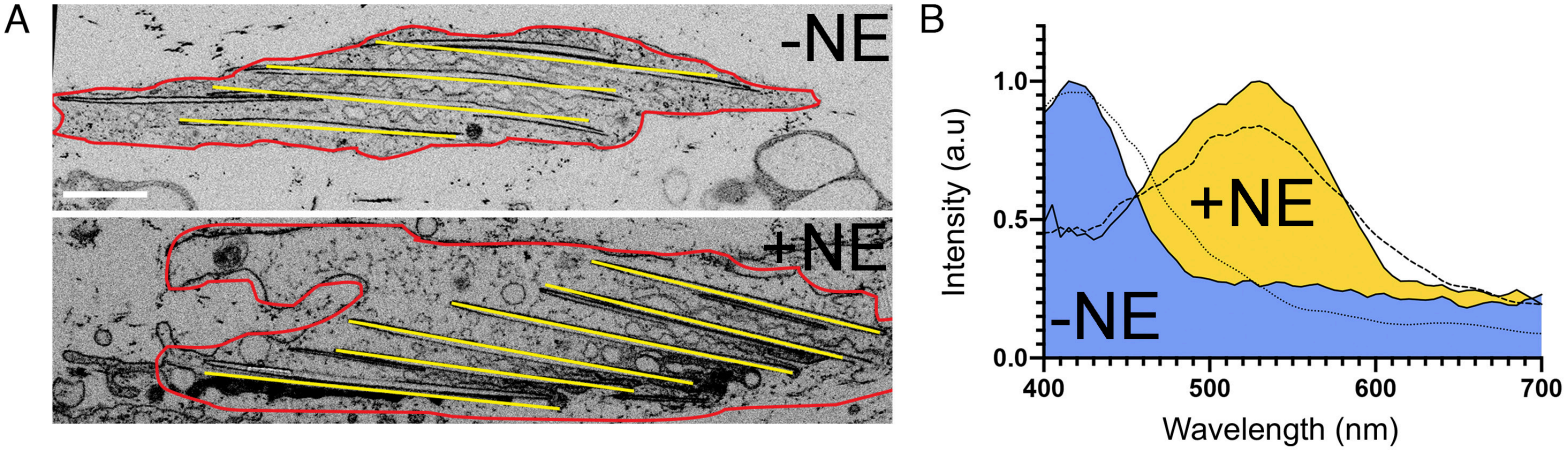
NE-induced changes in crystal tilt, crystal spacing, and cellular optical properties. (*A*) *Top* panel: representative FIB-SEM section (XZ plane) of an untreated iridophore. *Bottom* panel; representative FIB-SEM section (XZ plane) of a NE-treated iridophore. (*B*) Optical properties of untreated (blue curve) and NE-treated iridophores (yellow curve) compared to the corresponding Monte Carlo simulations (dotted for untreated, dashed for NE treated). (Scale bar, (*A*) 500 µm.)

Iridophore color is based on the constructive interference of light reflected from crystals with a particular spacing and orientation relative to the light. We hypothesized, therefore, that the change in the color of scales induced by NE is caused by one or more nanometer-sized changes in the organization of the crystals within scale iridophores. We used FIB-SEM of untreated and NE-treated scales to look for possible changes in crystal organization within scale iridophores. Two major differences in crystal organization were observed. First, the tilt in the angle of crystals relative to the surface of the scale increased from 13° ± 6° (n = 112) in untreated scales to 34° ± 8° (n = 142) in NE-treated scales (Fig. 2*A* and *SI Appendix*, Fig. S1). Second, the spacing between crystals increased from 120 nm ± 28 nm, (n = 167) in untreated scales to 165 nm ± 25 nm (n = 142) in NE-treated scales (Fig. 2*A* and *SI Appendix*, Fig. S1). In addition, in the treated scales the crystals were often not fully aligned. To determine whether these changes in crystal organization could explain the change in scale color seen upon NE addition, we compared the reflectance spectra of untreated and treated iridophores obtained using hyperspectral imaging microscopy to Monte Carlo simulations based on the morphometric data derived from the FIB-SEM data. We note that the simulations were conducted assuming that the crystal interfaces are parallel in both treated and untreated scales due to the limitations of the simulations. Notably, we found an excellent match between the cellular reflectance data and the simulations (Fig. 2*B*). This match argues that the two NE-induced changes in crystal organization seen using FIB-SEM, i.e., higher tilt angle and larger spacing, are indeed responsible for the observed color change in scales following NE addition.

**Fig. 3.**
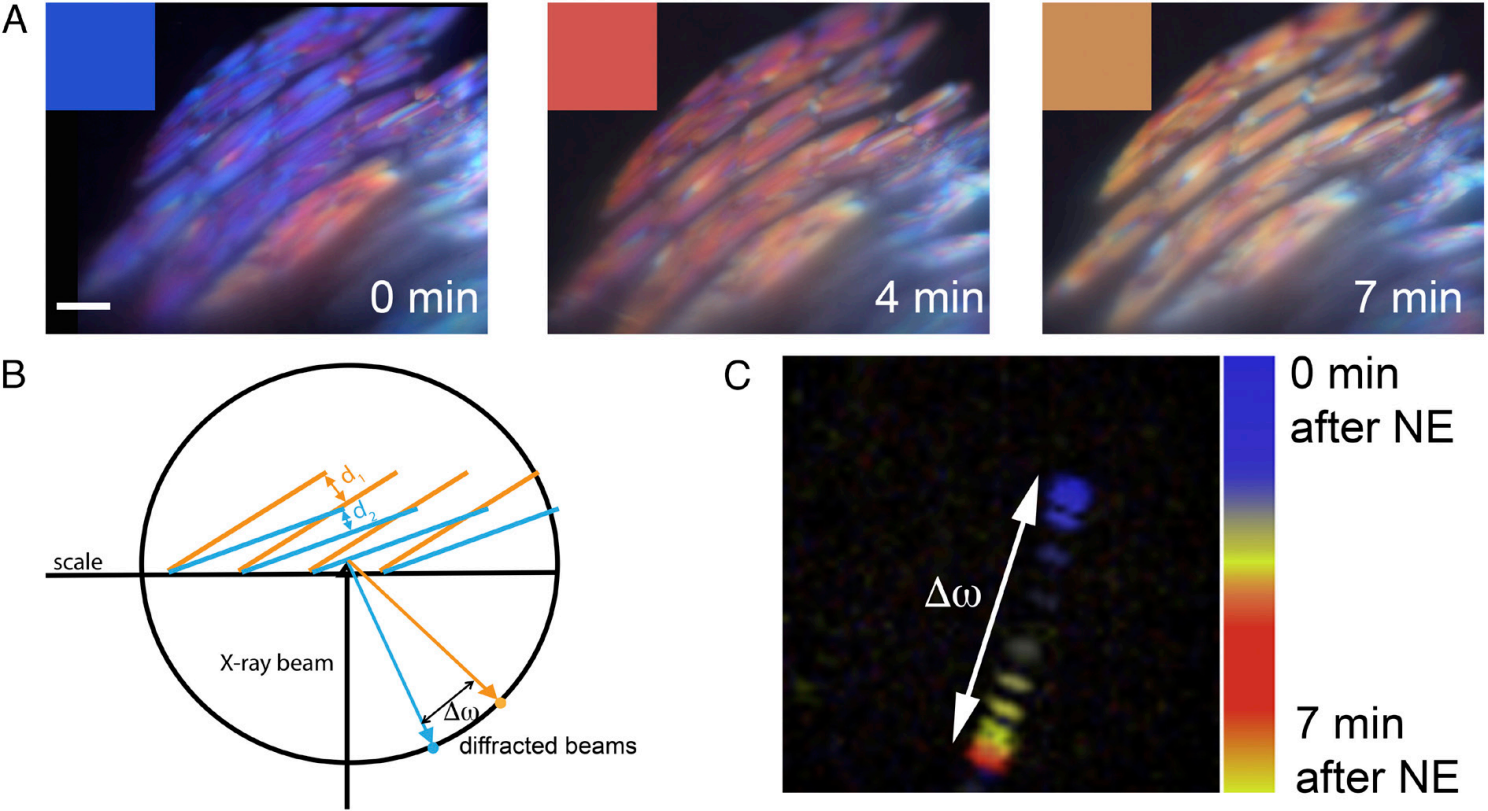
Color change in scale iridophores is driven by the tilting of crystals. (*A*) Iridophores are typically blue before NE treatment (*Left*), red 4 min after NE addition (*Middle*), and finally yellow 7 min after NE addition (*Right*) (these two shifts were seen in 6 out of 10 scales from three different fish). *Insets* show representative colors for each time point. (*B*) A schematic showing the X-ray diffraction experimental setup, where a change in the tilt orientation of crystals results in a change in the (Δω) of the diffraction spot. (*C*) Pseudotemporal colored representation of 32 diffraction spots over a 7-min period following NE addition. (Scale bar, (*A*) 10 µm.)

To investigate the relationship between crystal tilt and scale color in more detail, we determined the dynamics of iridophore color change in response to NE by time-lapse imaging with incident illumination (Fig. 3*A*). Intriguingly, about half of the iridophores first changed their color from blue (Fig. 3 *A*, *Left*, time 0) to red (Fig. 3 *A*, *Middle*, Time 4 min), and only then back to yellow, the final color (Fig. 3 *A*, *Right*, Time 7 min). If crystal tilting drives color change, then the change in crystal tilt that causes the blue to red shift should partially reverse during the final shift from red to yellow. To test this, we used synchrotron-based micro-X-ray diffraction to dynamically track the orientations of crystals at the single cell level following NE addition. If the crystals change their tilt, the azimuthal angle (ω) of the crystal diffraction spots will rotate by (Δω) (Fig. 3*B*). If the tilting reverses, this rotation will occur in the opposite direction. As predicted, the diffraction spot from a scale iridophore following NE addition first rotated clockwise for 5 min during the blue to red shift, and then rotated counterclockwise for 2 min during the final red to yellow shift (Fig. 3*C* and Movie S3). This close correlation between crystal tilt reversal and the fine-tuning of final scale color provides strong additional support for the idea that the tilting of the crystals in response to NE drives the NE-induced change in scale color.

### Crystals Move Bidirectionally in Untreated Iridophores, Unidirectionally in NE-Treated Iridophores, and Are Immobilized in Nocodazole-Treated Iridophores.

**Fig. 4.**
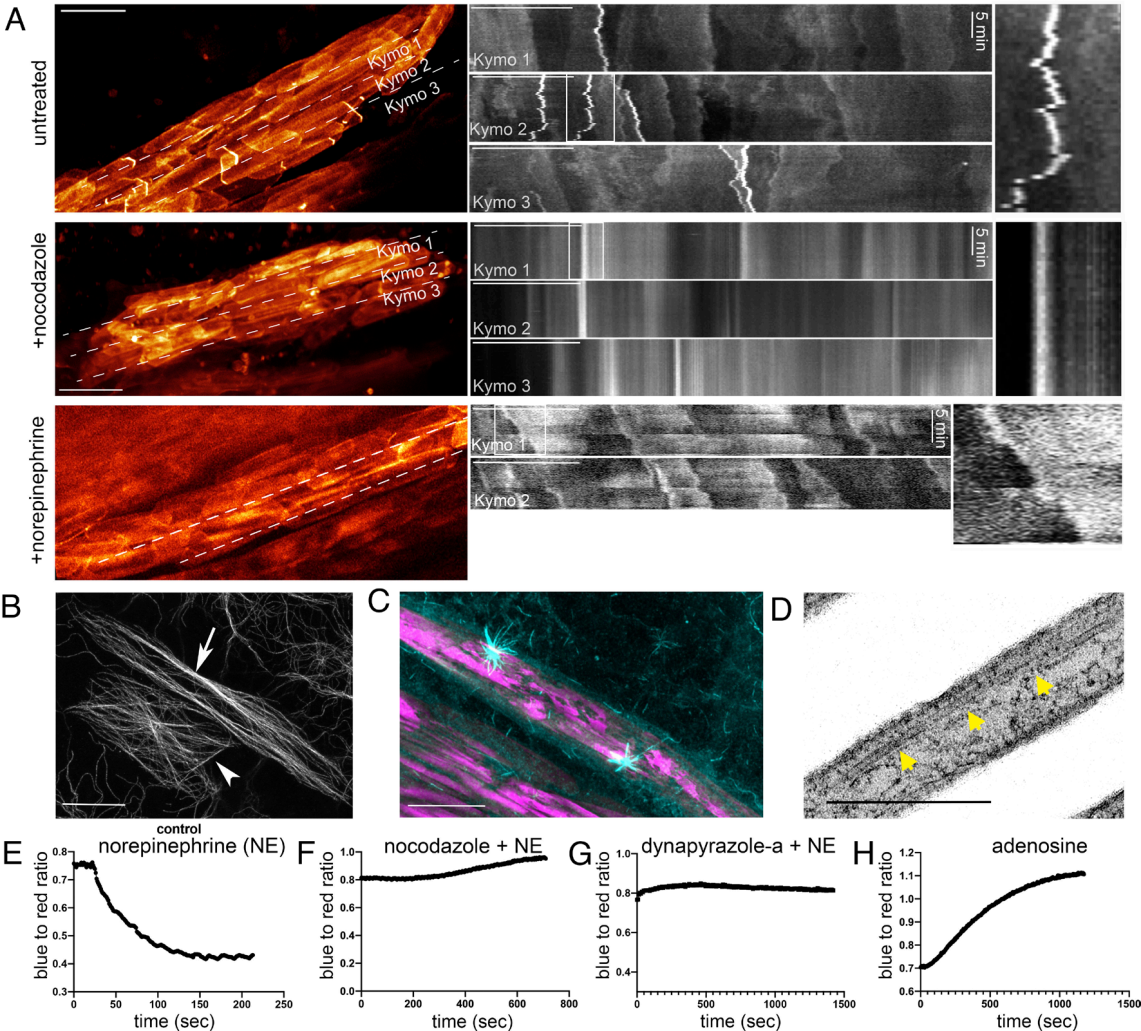
Microtubules and the microtubule minus end-directed motor protein dynein drive NE-induced color change. (*A*) JF549-labeled iridophores (*Left*) and corresponding kymographs of untreated (*Top*), nocodazole-treated (*Middle*), and NE-treated cells (*Bottom*). Kymograph *Insets* are contrasted for clarity. (*B*) Microtubule distribution visualized using an anti-α-tubulin antibody in an iridophore (arrow) and an adjacent dermal cell (arrowhead). (*C*) Iridophore in a scale after 10 min of recovery from nocodazole treatment (anti-α-tubulin, cyan; crystals autofluorescence, magenta). (*D*) TEM image of an iridophore showing ~25 nm tubes running parallel to the crystals. (*E*–*H*) Graphs showing the blue-to-red channel ratio (the deconvoluted components of an RGB movie) of iridophores treated with NE (*E*), nocodazole + NE (*F*), dynapyrazole-a + NE (*G*) and adenosine (*H*). (Scale bars, (*A*, *Left*) 4 µm, (*A*, *Right*) 10 µm, (*B*) 8 µm, (*C*) 5 µm, (*D*) 400 nm.)

We reasoned that the ability to image crystal dynamics in living iridophores by light microscopy would facilitate efforts to identify the cellular mechanism that drives crystal tilting in response to NE. We sought, therefore, to identify a dye that labels crystals brightly in living cells. While Malachite Green binds to crystals and can be used to visualize them statically in cells (33), we found that it is not sufficiently bright to track crystal movements in living cells over time. To identify a suitable dye, we screened multiple Janelia Fluor (JF) rhodamine derivatives for crystal binding and specificity, as they are structurally related to Malachite Green. Azetidinyl rhodamine (JF549) showed the best overall results (*SI Appendix*, Fig. S2). Using JF549 to stain crystals, we imaged otherwise untreated iridophores by Zeiss AiryScan (see *Materials and Methods* for a full description) to track crystal movements over time (note that JF549 stains crystals most intensely at their free ends). As anticipated, the crystals were seen to align with the cell’s long axis. Rather unexpectedly, crystals were also seen to move continuously in a back-and-forth fashion over distances of ~0.3 µm (Movie S4). This oscillatory motion is most evident when the intense staining at crystal ends is presented in kymograph form (Fig. 4 *A*, *Top*).

Given that membrane-bound organelles often move along microtubules, we asked whether crystal motions are microtubule dependent. To determine the distribution of microtubules within iridophores, as well as their orientation with respect to the guanine crystals, we fixed and stained isolated, wild-type scales with the anti-α-tubulin antibody DM1α (conjugated to an Alexa Fluor® 555). The resulting images show that microtubules span the entire cell and that they are oriented largely parallel to both the cell’s long axis and the elongated guanine crystals (Fig. 4*B*, Arrow and *SI Appendix*, Fig. S3 *A–D*). Of note, this microtubule organization is very different from that seen in neighboring pigment-producing melanophores, which exhibit a radial array of microtubules that emanate from a single microtubule organizing center (MTOC) (Fig. 4*B*, Arrowhead and *SI Appendix*, Fig. S3 *B* and *C*).

We next sought to identify the position of the MTOC(s) in iridophores, as this would provide insight into the polarity of the microtubule network. To accomplish this, we depolymerized microtubules using nocodazole and then fixed and stained the cells for tubulin at various times after nocodazole washout. Short recovery times (~10 min) consistently revealed two MTOCs per cell, with one near each end of the elongated cell and with the two being on opposite sides of the cell’s short axis (Fig. 4*C*). Longer recovery times revealed repolymerized microtubules extending from the two MTOCs toward the ends of the cell, forming an elaborate array parallel to the cell’s long axis, as observed in untreated cells (*SI Appendix*, Fig. S4). Importantly, this microtubule organization positions the minus ends of all the microtubules in the iridophore close to its two ends. Finally, high-resolution transmission electron microscopy (TEM; see *Materials and Methods* for a full description) confirmed that microtubules run parallel to and in between the crystals (Fig. 4*D*)**.**

Considering the close association between membrane-bound crystals and microtubules, we hypothesized that microtubules play a key role in driving NE-induced crystal movement. We asked, therefore, whether depolymerizing microtubules would impede crystal movement. Indeed, when we depolymerized microtubules using nocodazole, the translocation of the crystals was drastically reduced (Fig. 4 *A*, *Middle* and Movie S5). The reduced movement of nocodazole-treated cells was also evident from the differences observed in the distributions of crystal instantaneous speeds (*SI Appendix*, Fig. S5*A*). When crystals were moving consistently in the same direction, we measured speeds of up to 500 nm/s (*SI Appendix*, Fig. S5*B*). We also asked whether iridophore color change involves a change in the pattern of crystal motility. To test this, we treated cells with NE and followed crystal motility. Upon NE treatment, the previously observed oscillatory “back-and-forth” movement pattern was not detected and, instead, crystals moved consistently in the same direction (Fig. 4 *A*, *Bottom*).

### The Microtubule Minus End-Directed Motor Protein Dynein Drives NE-Induced Color Change.

The fact that microtubules run parallel to and in between crystals, and that crystals do not move in nocodazole-treated cells (Fig. 4*A*), suggests that microtubules play a central role in the crystal motions underlying color change. One possibility is that crystals are moved by forces placed on them by growing and/or shrinking microtubules. Alternatively, crystals might be moved by a microtubule motor protein attached to the crystal chamber membrane. Regarding this latter possibility, we chose to target the microtubule minus end-directed motor protein dynein in part because the minus ends of all microtubules in the iridophore reside close to the two ends of the cell’s long axis (Fig. 4*C*). It is also the case that the best small molecule inhibitors of microtubule motor proteins are all against dynein [e.g., ref. 34 and dynapyrazole-a (35)]. To access the relative contributions of microtubule dynamics and dynein to color change, we quantified NE-induced color change in iridophores (Fig. 4*E*) treated with either nocodazole (Fig. 4*F*) or dynapyrazole-a (Fig. 4*G*) by deconvoluting time-lapse images into their RGB components and plotting the ratio of blue-to-red channels. If microtubule dynamics are solely responsible for moving the crystals, then nocodazole should block color change and dynapyrazole-a should have little to no effect. If, on the other hand, dynein is contributing to color change, then both nocodazole and dynapyrazole-a should inhibit color change. As expected, control iridophores treated with NE changed their color within a few minutes, as manifested by a drop in the blue-to-red ratio (Fig. 4*E*). Importantly, iridophores that were pretreated with either nocodazole (Fig. 4*F*) or dynapyrazole-a (Fig. 4*G*) failed to change color in response to NE. This result indicates that microtubule dynamics cannot by itself be driving crystal motion, and that dynein likely drives crystal motion/color change by moving crystals toward microtubule minus ends.

While observing scale iridophores as they change color in response to NE, we noticed that the size of the gap separating neighboring iridophores shrinks over time, consistent with a progressive increase in cell volume. Relevant to this observation, a decrease in the osmolarity of a medium can induce color changes in fish (24). Given this, we asked whether the NE-induced color change we studied here is caused by a change in cell volume. Simultaneous measurements of cell area and cell color in individual cells responding to NE showed that iridophore cell area increases on average by ~25%, and that this change precedes a gradual change in color (*SI Appendix*, Fig. S6). Importantly, cells treated with nocodazole prior to NE treatment still show this ~25% increase in size, but they do not change color. This result argues that the change in cell volume induced by NE is not responsible for the change in cell color.

### Cellular cAMP Likely Regulates Structural Color Change in Iridophores.

Classic studies of NE-induced changes in pigment-based coloration of fish and amphibians have shown that NE addition leads to a decrease in the level of cAMP within melanophores (9, 36–40). Importantly, this decrease promotes the activation of cytoplasmic dynein on the melanophore’s pigment granules, causing their translocation toward microtubule minus ends. Because all of these ends are anchored at the single MTOC in the center of the melanophore, the end result of NE addition is the aggregation of the pigment granules in the center of the cell. Consistently, NE addition leads to the aggregation of pigment granules in zebrafish melanophores (33, 41, 42). Given this, and given our evidence here that NE addition drives structural color change in iridophores by promoting the dynein-dependent tilting of crystals, we asked whether the ability of NE to drive the shift from blue to yellow could be antagonized by the addition of a factor to the media that causes a rise in intracellular cAMP. To accomplish this, we pretreated scales with NE for 10 min, washed the NE out, and then treated the scales with adenosine. Adenosine is a cell-permeable molecule that causes a rapid increase in intracellular cAMP levels by activating adenyl cyclase (43, 44) While the control scales maintained their yellow color for more than one hour (*SI Appendix*, Fig. S7), scales treated with adenosine changed their color back to blue within 10 min (Fig. 4*H* and Movie S6). A similar effect was seen when dibutyryl-cAMP, a cell-permeable cAMP analog, was added to NE-treated scales (*SI Appendix*, Fig. S8). Together, these results argue that the NE-induced, dynein-dependent tilting of crystals in iridophores, like the NE-induced, dynein-dependent aggregation of pigment granules in melanophores, is regulated by cAMP.

## Discussion

While changes in structural colors are known to be used by animals for many purposes (e.g., to hide from predators, to communicate, to regulate body temperature), the cellular mechanisms that drive structural color change are largely unknown. Here we used zebrafish scale iridophores to uncover one such mechanism. Using FIB-SEM-based serial sectioning and micro-focused X-ray diffraction, we showed that in response to stress the iridophore’s guanine crystals tilt by ~20°, which increases the spacing between the crystals by ~40 nm. This change in crystal spacing results in different constructively interfering wavelengths, changing the iridophore’s color from blue to yellow. Using immunostaining for microtubules, a dye to image crystal dynamics, and several pharmacological perturbations, we presented evidence that the microtubule minus end-directed motor dynein drives the NE-induced change in crystal angle by binding to the crystal chamber limiting membrane and walking toward microtubule minus ends. It should be noted, however, that at present we cannot distinguish between regulated binding of dynein to the crystal chamber membrane, activation of dynein already on the membrane, and enhancement of the microtubule track by posttranslational modification as contributors to the NE-induced change in crystal angle. Indeed, some combination of all three mechanisms may be at play. Finally, we obtained evidence that cytoplasmic cAMP levels regulate this process.

The color reflected from neighboring iridophores is uniform, suggesting the involvement of intercellular communication and regulation. A potential manifestation of this regulation is the observation that about half of the cells we monitored during color change “overshot” their final color, changing initially from blue to red and then “fine-tuning” back to yellow. This response of isolated iridophores to NE also indicates that they have the intrinsic ability to sense NE levels in their environment. Indeed, androgenic receptors, the cellular receptors of NE, were recently shown to be highly expressed in the motile iridophores of zebrafish blue stripes (33).

Previous work in other pigment cells has shown that the binding of NE to androgenic receptors decreases the level of cAMP in the cytoplasm (38–40, 45). Our findings that an increase in intracellular cAMP, induced by treating cells with either adenosine or dibutyryl-cAMP, reverses the effect of NE argues that cAMP is involved in regulating structural color change in iridophores. cAMP is known to downregulate the activity of the cytoplasmic protein kinase A (PKA) (9). Moreover, a recent study showed that PKA phosphorylates cytoplasmic dynein, and that this modification inhibits dynein’s interaction with specific organelles, impeding their transport (46, 47). It is thus reasonable to think that the response of iridophores to stress, which begins with the binding of NE, eventually leads to the activation of cytoplasmic dynein and the microtubule minus end-directed movement of the membrane-bound crystals. Of note, we also found that NE leads to an increase in iridophore cell volume, and that this increase precedes color change and occurs even when crystal movement is blocked by depolarizing microtubules. These observations argue that the change in cell volume induced by NE is not responsible for the change in cell color, and that the volume increase is either upstream of crystal tilting or occurs via a separate cellular cascade. With regard to water uptake by iridophores, a recent study showed that several aquaporins, which facilitate water transport into cells, are highly expressed in zebrafish iridophores and may be involved in this process (33).

The exact mechanism by which the dynein-dependent movement of crystal chambers drives the tilting of the crystals required for iridophore color change remains to be clarified. Our FIB-SEM data showed that the crystal chambers are parallel to one another both before and after color change. Therefore, direct interactions between dynein and membrane-bound crystals could underlie the tilting. In this scenario, the microtubule minus end-directed movement of crystal chambers along microtubules aligned parallel to the cell’s long axis would tilt the crystal-containing organelles in the same direction to drive color change. Regarding the force that is responsible for restoring the angle of the crystals to their pre-NE position, while a plus end-directed microtubule motor could be involved, we suspect that elastic forces are actually responsible for restoration. Future work will be required to answer this question.

In conclusion, we have shown that the microtubule minus end-directed motor protein dynein is required for NE-induced crystal tilting and consequent color change in zebrafish iridophores. This previously unexplored cellular response allows iridophores to change the spacing between their guanine crystals in a controlled and uniform manner, altering the pattern of coherent light reflection and thus color emission from the iridophore. We propose that this cellular response involves interactions between the membrane surrounding the guanine crystals, dynein, and microtubules, and that these interactions lead to the directed movement of the guanine-crystal encased membrane, which, in turn, changes the angle of reflected light from the crystals. This mechanism may be used by other organisms that exhibit structural color change and could inspire the construction of artificial, tunable photonic crystals.

## Materials and Methods

### Zebrafish Husbandry and Handling.

Zebrafish (*Danio rerio*) were housed at ∼28 °C with a 14 h light, 10 h dark cycle, and fed with Artemia and flake food. Fish were maintained and fed following standard protocols.

### Scale Extraction and Drug Treatments.

Scales were extracted from anesthetized, wild-type adult zebrafish and were placed in 0.66× PBS solution to maintain an osmolarity of approximately 200 mOsm/kg. Scales were then extracted and flipped upside down to reveal iridophores and mounted on slides for live imaging. When scales were treated with norepinephrine (Sigma, A7257), nocodazole (Sigma, M1404), adenosine (Sigma, A9251), forsoklin (Sigma, F6886), or dynapyrazole-a (Sigma, SML2127) a final concentration of 10 µM was used. Scales used for micro-Raman spectroscopy or confocal live imaging were embedded in 0.9% low-melt agarose on a glass-bottom dish, and covered with 0.66× PBS.

### Confocal Live Cell Imaging and Immunohistochemistry.

Airyscan imaging of iridophores was carried out on an inverted Zeiss LSM 880 using a C-apochromat 40×/1.2NA autocorr M27 water objective. Samples were illuminated with 561 nm and 633 nm laser lines. Airyscan processing was performed in Zen Black (Zeiss). Cells were fixed in 4% paraformaldehyde/0.1% glutaraldehyde (Electron Microscopy Services) and permeabilized with Triton-X 100 (0.05%) to image crystals and microtubules simultaneously using AlexaFluor555-Dm1α (Sigma, 05-829X-555) at a concentration of 1:100. Cells were fixed in ice-cold methanol when examining microtubules alone. The live cell dye JF_525_-Tubulin was kindly provided by Luke D. Lavis’s lab and used at a concentration of 10 µM.

### Image Processing.

Crystal instantaneous speed were measured by manual tracking of individual crystals using the Fiji plugin TrackMate (48). The kymographs in Fig. 4*A* were generated in Fiji using the “Reslice” tool along the indicated line scans. For assessing microtubule orientation, images were processed in Fiji. The depth color-coded maximum-intensity projections of iridophore microtubules (*SI Appendix*, Fig. S3*D*) were generated using the “Temporal-Color Code” function in Fiji. Microtubules orientation analyses (*SI Appendix*, Fig. S3 *E–G*): Binary microtubule skeletons of 11 dermal cells and 11 iridophores were generated in Fiji using a ridge (49). Microtubule skeleton orientations were calculated using the OrientationJ Fiji (50). Microtubule resultant vector lengths and mean directions were calculated using the MATLAB CircStat (51). Ridgeline plots (*SI Appendix*, Fig. S3 *F* and *G*) indicate the angular dispersion of all microtubules relative to the mean direction of the microtubule network in each cell. Plots are pseudocolored by resultant vector length, where 1 indicates maximally nonuniform microtubule distribution (i.e., perfect co-orientation) and values closer to 0 indicate increased microtubule dispersion.

Color change analyses were performed using a custom MATLAB code that deconvolves RGB image series into its different compensates and sums the intensities for each channel over the entire image. The ratio of the blue to red channels was then plotted as a function of time.

### Crystal Dye Identification for Staining Crystals.

To identify a suitable dye, multiple Janelia Fluor (JF) rhodamine derivatives they are structurally related to Malachite Green were screened for crystal binding and specificity. All dyes used in this work were kindly provided by Luke D. Lavis’s lab and include JF_549_, JF_519_, and JF_646_, which were originally screened and published by Grimm et al. (52); JF_585_ and JF_635_ by Grimm et al. (53); JF_526_ by Zheng et al. (54); JF_614_, JF_630_, and JF_639_ Deo et al. (55).

### FIB-SEM.

Zebrafish scales were divided into two groups (NE-treated and untreated) and processed similarly. For high pressure freezing, the scales were rinsed in 0.66× PBS, dipped in hexadecane, and placed within a 100 µm well of a type “A” specimen carrier (Technotrade international # 241) (treated with hexadecane). The flat side of the type “B” 300 µm thick specimen carrier (Technotrade international #242) (treated with hexadecane) was placed over the type “A” carrier to sandwich the scale, making sure to avoid air bubbles. The sandwich was then secured within the holder and plunge frozen in liquid propane using a Wohlwend Compact 1 HPF. The frozen sandwiches were separated under liquid nitrogen and transferred into cryovials containing a freeze substitution cocktail of 2% OsO4, 0.1% UA, 3% H_2_O in acetone. The freeze substitution protocol was as follows: 140 °C to −90 °C over 2 h; −90 °C to −90 °C over 24 h; −90 °C to 0 °C over 12 h; 0 °C to 22 °C over 1 h; 22 °C hold for 2 h. After freeze substitution, samples were rinsed 3 × 10 min with pure 100% Acetone. Infiltration was performed at room temperature with increasing concentrations of DurcupanACM (EMS catalog # 14040) in Acetone as follows: Durcupan: Acetone, 1:2 for 1 h; 1:1 for 2 h; 2:1 for 2 h; overnight in 100% Durcupan, followed by two additional changes in 100% Durcupan with an accelerator at 3 h intervals before placing in a flat silicone embedding mold and polymerizing for 48 to 72 h at 60 °C.

Durcupan-embedded zebrafish scales (NE-treated and untreated) were then mounted on top of a 1 mm copper post that was in contact with the metal-stained sample for belter charge dissipation, as previously (56). The vertical sample posts were then trimmed to a small block containing the region of interest (ROI) with a width perpendicular to the ion beam, and a depth in the direction of the ion beam. The block sizes were 150 × 60 µm^2^ and 115 × 60 µm^2^ for untreated and NE-treated scales, respectively. The trimming was guided by X-ray tomography data obtained using a Zeiss Versa XRM-510 and optical inspection under a microtome. The trimmed samples were then coated with thin layers of conductive material made of 10 nm gold followed by 100 nm carbon using a Gatan 681 High-Resolution Ion Beam Coater. The coating parameters were 6 keV, 200 nA on both argon gas plasma sources and 10 rpm sample rotation with a 45-degree tilt.

FIB-SEM-prepared samples were imaged sequentially using a customized Zeiss FIB-SEM system described previously (56–58). The block face of the ROI was imaged using a 2 nA electron beam with 1.2 keV landing energy at a 2 MHz scanning rate. The x-y pixel resolution was set at 8 nm. A subsequently applied focused Ga+ beam of 15 nA at 30 keV strafed across the top surface and ablated away 8 nm of the surface. The newly exposed surface was then imaged again. The ablation imaging cycle continued about once every 45 s for 12 d to complete FIB-SEM imaging of the untreated sample, and about once every minute for 10 d to complete the NE-treated sample. The acquired image stacks containing the raw imaged volumes were subjected to image registration and alignment using a Scale Invariant Feature Transform (SIFT) based algorithm. The aligned stack consists of a final isotropic volume of 120 × 30 × 204 µm^3^ and 100 × 50 × 118 µm^3^ for untreated and NE-treated samples, respectively. The voxel size of 8 × 8 × 8 nm^3^ was maintained for each sample throughout entire volumes, and can be viewed in any arbitrary orientations.

### Reflectivity Simulations.

The reflectivity spectrum of both untreated and NE-treated iridophores was simulated based on crystal thicknesses, orientation, and spacing obtained from FIB-SEM images using a Monte Carlo transfer matrix calculation, as described in detail in supporting information of Gur et al. (30). In brief, the reflectivity was calculated by averaging 700 runs in which each layer was characterized by its refractive index and layer thickness. The thickness of the layer was determined randomly based on the distributions derived from experimental data. This approach better captures the statistical ensemble rather than focusing on a specific instance, thereby facilitating a more generalized solution. The refractive index of the guanine crystal plates was taken as 1.83, which is the refractive index in the direction of the impinging light, and the refractive index of the cytosol was taken as 1.34. The weak dependence of the refractive index on wavelength was neglected, and we assumed that all the interfaces (i.e., inside a crystal stack and between stacks) were parallel. We also assumed no correlation between the crystal spacings within a single crystal stack.

### Reflectivity Measurements.

The reflectivity of both NE-treated and untreated fresh scales was measured using the Ocean insight FLAME mini-spectrophotometer attached to the DM6B microscope (Leica). A white diffuse reflectance standard (Labsphere USRS-99-010, AS-01158- 060) was used as reference.

### Synchrotron-Based Micro-Wide X-ray Diffraction.

Fish scales were immersed in a physiological buffer (PBS, P3813, Sigma-Aldrich, 136 mOsm/kg) and mounted on a lead tape between two Kapton windows. A small syringe connected to rubber-based capillary was inserted in between the Kapton window. In situ wide X-ray diffraction (WAXD) was obtained at the μ-Spot beamline, in the synchrotron radiation facility BESSY II, Helmholtz-Zentrum Berlin für Materialien und Energie, Berlin, Germany. Samples were mounted on a y–z scanning table and scans of the sample in areas of interest were performed. The microbeam was defined by a toroidal mirror and a pinhole of 30 μm diameter, providing a beam size of ~30 × 30 μm^2^ at the sample position. Time loop scans were performed on the scales with ~10 s interval before and after the addition of NE. An energy of 15 keV (λ = 0.82656 Å) was selected by a Mo/BC multilayer monochromator. The SAXS/WAXD patterns were measured using a MarMosaic 225 CCD-based area detector (Rayonix) placed at a sample–detector distance of 294 mm. The beam center in the detector and the sample–detector distance were calibrated using the powder X-ray diffraction pattern of a synthetic guanine powder. Data analysis and the integration of the 2D scattering patterns was performed using DPDAK55. The data were normalized with respect to the primary beam monitor (ionization chamber) and corrected for background caused by pinhole and air scattering.

### Micro-Raman Spectroscopy.

Micro-Raman spectroscopy (λ = 532 nm excitation) was collected using a Horiba LabRAM HR Evolution (Horiba) spectrometer equipped with four laser lines (325 nm, 532 nm, 633 nm, and 785 nm). The system has an 800 mm focal length spectrograph with several interchangeable gratings and was coupled to an open-electrode, front-illuminated, cooled CCD camera. The sample was placed under a modular microscope (Olympus BX-FM) with a suitable objective. For this work, LUMPlanFL N 60× NA = 1.0 W and MPlanFL N 150× NA = 0.9 BD (Olympus Japan) objectives with spatial resolutions better than 1 µm were used. The light from the Doubled Nd:YAG was dispersed on 600 and 1,800 g/mm grating and the pixel resolution was better than 2 cm^−1^, or about 0.5 cm^−1^, respectively. Spectra were normally collected between 100 to 1,800 cm^−1^, with a power of up to 2 mW and an exposure of 100 to 300 s using 2 to 5 averages. The data went through polynomial baseline subtraction and spike removal as is commonly done.

## Supplementary Material

Appendix 01 (PDF)

Movie S1. 3D reconstruction of a scale iridophore using FIB-SEM. This movie shows a high magnification view of the cell’s ultrastructure and the ~20-30 nm thick crystals running in parallel and aligned with the cell’s long axis (highlighted by pseudo coloring in blue and gray; the cell’s nucleus is pseudo colored red).

Movie S2. Detailed visualization of crystal arrangement and organization within iridophores using FIB-SEM. This movie shows a close-up view of the crystal arrays highlighted by pseudo coloring in blue and gray; the cell’s nucleus is pseudo colored red.

Movie S3. Synchrotron-based micro-X-ray diffraction of a scale iridophores following NE addition. In this movie the azimuthal angle (ω) of the diffraction spot of beta anhydrous guanine crystals located in the fish scales, were dynamically tracked by scanning with ~10 seconds interval after the addition of NE. This movie shows that following NE addition, the crystal diffraction spots rotated clockwise during the blue to red color change and counterclockwise during the final red to yellow color change. This indicates that the tilting of the crystals in response to NE drives the NE-induced change in scale color (i.e. that the change in the tilt orientation of the crystals is in sync with the observed color change).

Movie S4. Live imaging of iridophore crystal movements in a living iridophore stained with the JF549 dye, which labels the guanine crystals (note that JF549 stains crystals most intensely at their free ends). The crystals are seen to align with the cell’s long axis and move continuously in a back-and-forth fashion over distances of ~0.3 μm. The oscillatory motion is most evident when presented in kymograph form (Fig. 4A, Top).

Movie S5. Microtubule depolymerization drastically impedes crystal movement. The movie shows a nocodazole-treated iridophore stained with JF549 to visualize crystal movements. Compared to the control (Fig. 4*A* and *SI Appendix, Movie 5*), nocodazoletreated cells display a significant reduction in crystal movement, indicating that microtubules play a key role in driving crystal movement.

Movie S6. Time-lapse imaging of a zebrafish scale treated with adenosine following treatment with norepinephrine (NE). NE induces the tilting of iridophore crystals and shifts the color from blue to yellow, while adenosine antagonize the NE-induced color change by elevating intracellular cAMP levels. This movie shows that the addition of adenosine causes a rapid reversal of the color change, with the yellow color transitioning back to blue within 10 minutes of treatment. A similar trend was observed when treating cells with dibutyryl-cAMP, a cAMP analogue (*SI Appendix, Fig. S7*).

## Data Availability

All study data are included in the article and/or supporting information.
