# Supplementary material for: The physical and cellular mechanism of structural color change in zebrafish
Source: Proc Natl Acad Sci U S A. 2024 May 28;121(23):e2308531121. doi: 10.1073/pnas.2308531121 (PMC11161791; doi:10.1073/pnas.2308531121)
Supplement: Supplementary file 1 — Appendix 01 (PDF) [file pnas.2308531121.sapp.pdf]

PNAS

Supporting Information for

The physical and cellular mechanism of structural color change in zebrafish

Dvir Gur[1,2,3,*], Andrew Moore[2], Rachael Deis[1], Pang Song[2], Xufeng Wu[3], Iddo Pinkas[4], Claire Deo[2], Nirmala Iyer[2], Harald F. Hess[2], John A. Hammer[3,*], Jennifer Lippincott-Schwartz[*,2]

Corresponding authors:
Dvir Gur
Email: dvir.gur@weizmann.ac.il

John A. Hammer
Email: hammerj@nhlbi.nih.gov

Jennifer Lippincott-Schwartz
Email: lippincottschwartzj@janelia.hhmi.org

This PDF file includes:

Figures S1 to S8
Legends for Movies S1 to S6

Other supporting materials for this manuscript include the following:

Movies S1

Supplemental Figures

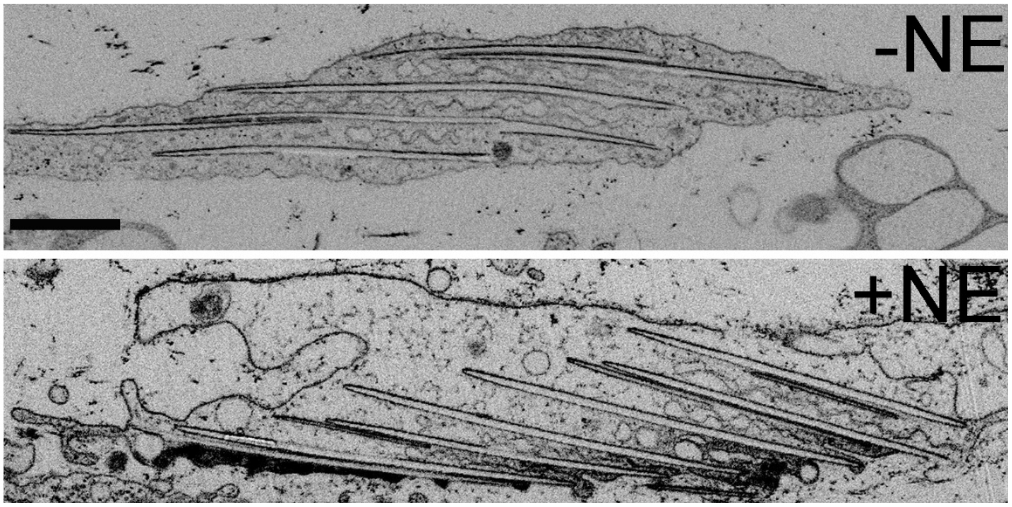

Supplemental Figure 1. Effects of NE Exposure on Iridophore Structure and Optical Characteristics. Top panel shows a cross-sectional view (XZ plane) of an untreated iridophore, and in the bottom panel, of an iridophore treated with NE. The scale bar is 500 µm.

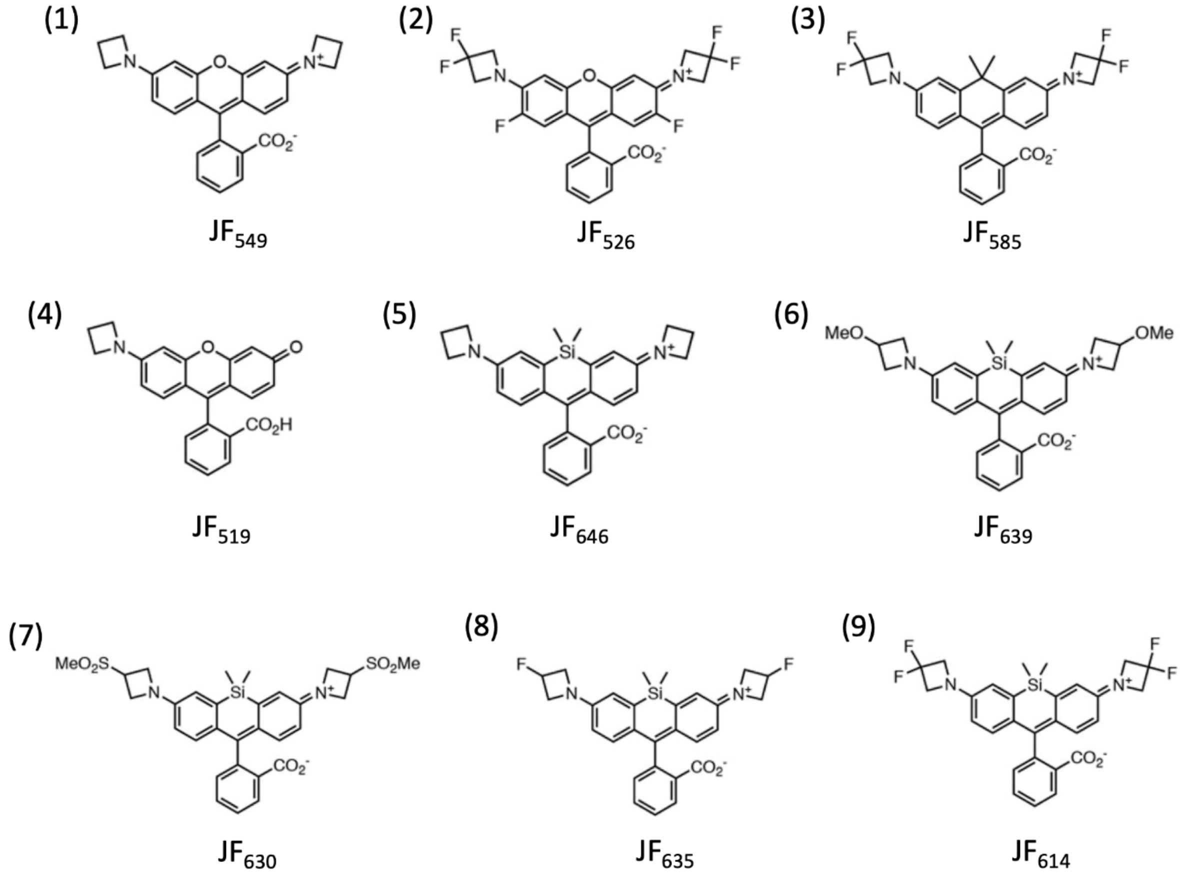

Supplemental Figure 2. Crystal dyes tested. JaneliaFluor (JF) rhodamine derivatives that were screened for crystal binding efficacy and specificity.

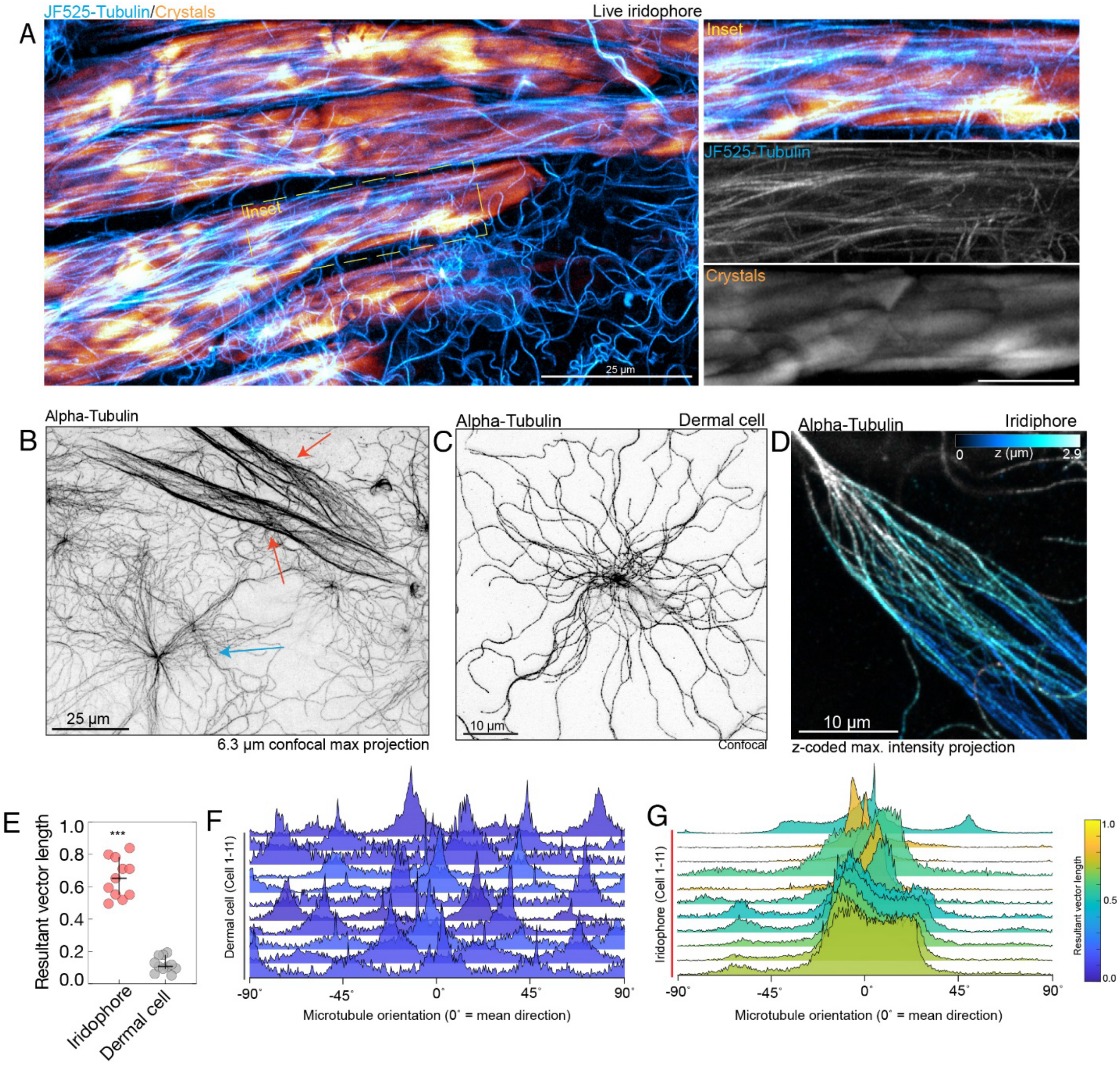

Supplemental Figure 3. Microtubule organization in iridophores. (**A**) Airyscan maximum intensity projection of crystals (autofluorescence) and microtubules (JF526-Tubulin) in live zebrafish scale iridophores. The yellow box marks the position of the magnified inset displayed on the right. Scale = 25 µm, 10 µm (inset). (**B**) Confocal maximum intensity projection of a fixed scale iridophore stained for microtubules (anti-alpha tubulin). The red arrows indicate the positions of the iridophores, and the blue arrow indicates the position of a neighboring dermal cell. Scale = 25 µm. (**C**) Confocal image of the microtubule network (alpha-tubulin) in a fixed dermal cell. Scale = 10 µm. (**D**) Airyscan color-coded maximum intensity projection of the tip of an iridophore. Scale = 10 µm. (**E**) Resultant vector length of microtubule orientations. A value of 1 indicates perfect alignment of all microtubules in the mean direction. Median ± interquartile range. n = 11 cells per condition. ***p<.001 unpaired t test. Ridgeline plot indicating the distribution of

microtubule orientations in 11 dermal cells (**F**) or iridophores (**G**). 0˚ indicates mean direction. Distributions are color coded by resultant vector length.

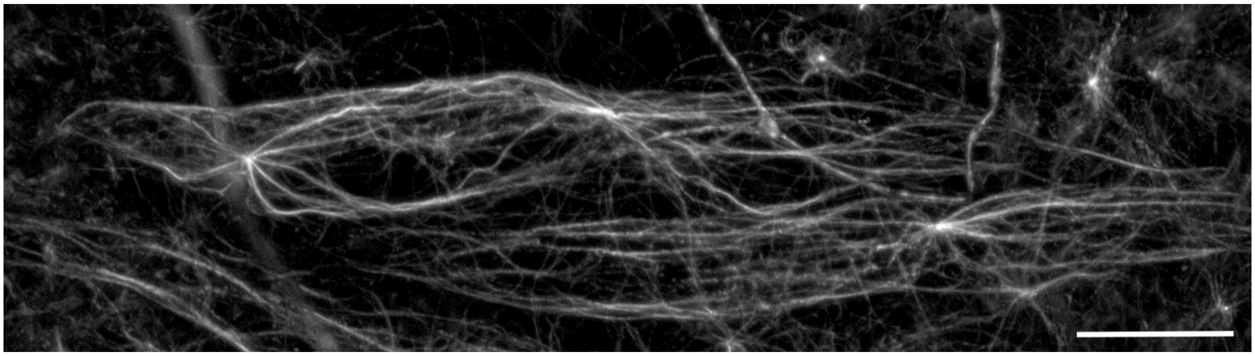

Supplemental Figure 4. Microtubule organization in iridophores following NZ washout. Immunostaining of iridophore microtubules with an anti-α-tubulin antibody (DM1A, Alexa Fluor® 555-conjugated) 55 min after nocodazole washout shows that they form arrays parallel to the cell's long axis just as in untreated cells. Scale = 20 μm.

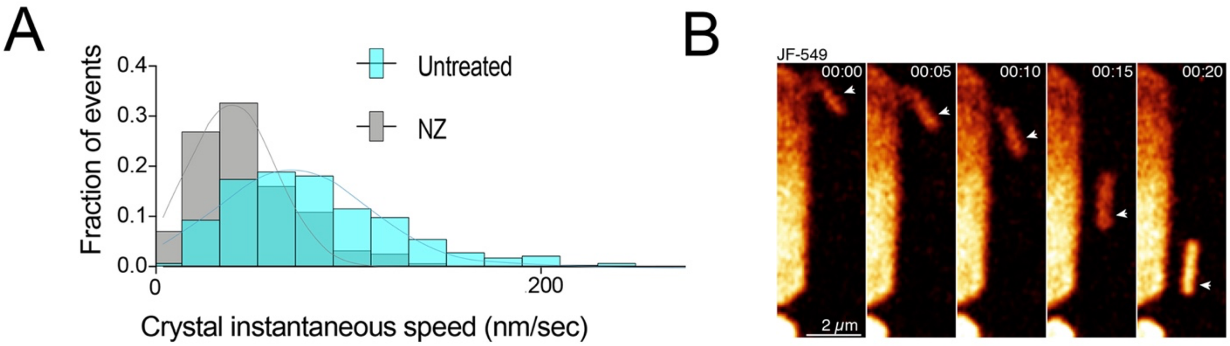

Supplemental Figure 5. Crystal instantaneous speeds. (**A**) Shown are the distributions of crystal instantaneous speeds for untreated scales (cyan) and scales treated with nocodazole (gray). (**B**) A crystal moving consistently in the same direction with speeds of up to 500 nm/\sec.

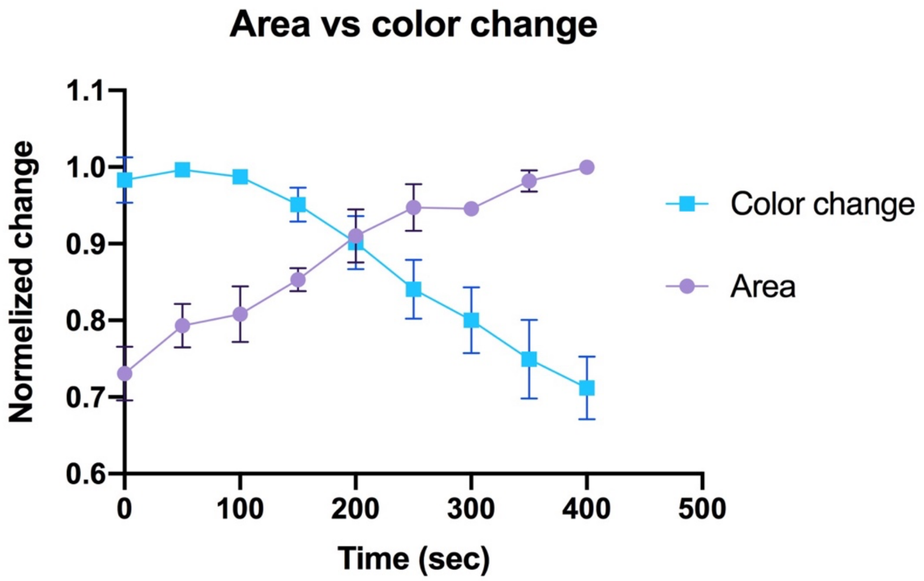

Supplemental Figure 6. The color change (blue to red ratio) vs the area change measured for individual iridophores upon administration of NE. The change in area for treated cells individually tracked was on average ~20% (purple curve). This change in area preceded the color change by 60-100 sec (light blue curve). n=27 cells, from 3 scales taken from 3 different adult fish.

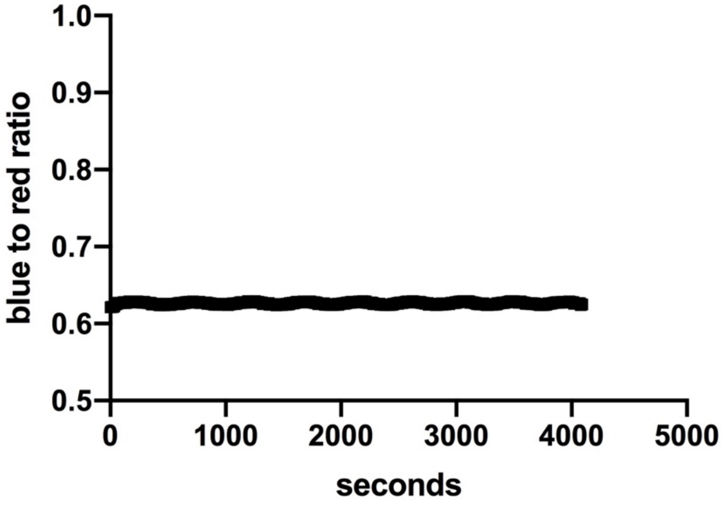

Supplemental Figure 7. Scale iridophores pretreated with NE maintain their yellow color after treatment. Treated cells, which change their color to yellow upon administration of NE, maintain their yellow color and did not change their color back to blue if not treated with a cAMP analogue (e,g. dibutyryl-cAMP).

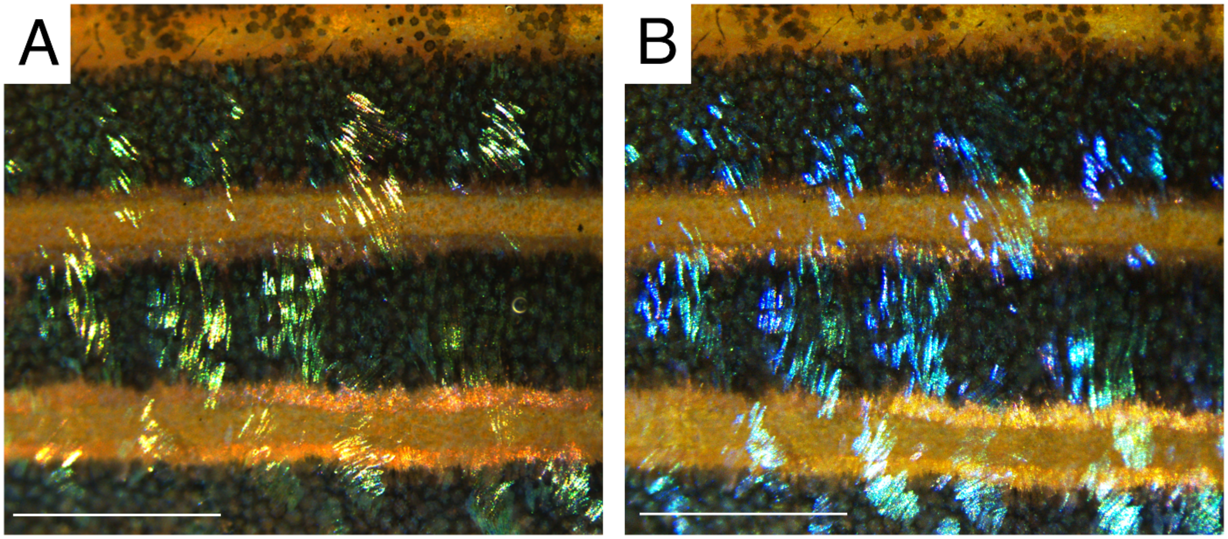

Supplemental Figure 8. NE-induced, dynein-dependent tilting of crystals in iridophores is regulated by cAMP. Control scales remained yellow colored for more than one hour (**A**) while scales treated with dibutyryl-cAMP, a cell-permeable cAMP analog, changed their color back to blue in 10 minutes (**B**). Scale bar 0.5 mm.

Movies

Movie S1: 3D reconstruction of a scale iridophore using FIB-SEM. This movie shows a high magnification view of the cell's ultrastructure and the ~20-30 nm thick crystals running in parallel and aligned with the cell's long axis (highlighted by pseudo coloring in blue and gray; the cell's nucleus is pseudo colored red).

Movie S2: Detailed visualization of crystal arrangement and organization within iridophores using FIB-SEM. This movie shows a close-up view of the crystal arrays highlighted by pseudo coloring in blue and gray; the cell's nucleus is pseudo colored red.

Movie S3: Synchrotron-based micro-X-ray diffraction of a scale iridophores following NE addition. In this movie the azimuthal angle (ω) of the diffraction spot of beta anhydrous guanine crystals located in the fish scales, were dynamically tracked by scanning with ~10 seconds interval after the addition of NE. This movie shows that following NE addition, the crystal diffraction spots rotated clockwise during the blue to red color change and counterclockwise during the final red to yellow color change. This indicates that the tilting of the crystals in response to NE drives the NE-induced change in scale color (i.e. that the change in the tilt orientation of the crystals is in sync with the observed color change).

Movie S4: Live imaging of iridophore crystal movements in a living iridophore stained with the JF549 dye, which labels the guanine crystals (note that JF549 stains crystals most intensely at their free ends). The crystals are seen to align with the cell's long axis and move continuously in a back-and-forth fashion over distances of ~0.3 µm. The oscillatory motion is most evident when presented in kymograph form (Fig. 4A, Top).

Movie S5: Microtubule depolymerization drastically impedes crystal movement**.** The movie shows a nocodazole-treated iridophore stained with JF549 to visualize crystal movements. Compared to the control (Fig. 4*A* and *SI Appendix, Movie 5*), nocodazole-treated cells display a significant reduction in crystal movement, indicating that microtubules play a key role in driving crystal movement.

Movie S6: Time-lapse imaging of a zebrafish scale treated with adenosine following treatment with norepinephrine (NE). NE induces the tilting of iridophore crystals and shifts the color from blue to yellow, while adenosine antagonize the NE-induced color change by elevating intracellular cAMP levels. This movie shows that the addition of adenosine causes a rapid reversal of the color change, with the yellow color transitioning back to blue within 10 minutes of treatment. A similar trend was observed when treating cells with dibutyryl-cAMP, a cAMP analogue (*SI Appendix,* Fig. S7).
